# Pomegranate peel as a phytogenic in broiler chickens: Influence upon antioxidant, lipogenesis and hypotensive response

**DOI:** 10.1002/vms3.556

**Published:** 2021-06-15

**Authors:** Behnam Ahmadipour, Sajad Pat, Samira Abaszadeh, Hossein Hassanpour, Fariborz Khajali

**Affiliations:** ^1^ Faculty of Agriculture Department of Animal Science Shahrekord University Shahrekord Iran; ^2^ Faculty of Veterinary Medicine, Department of Basic Sciences Shahrekord University Shahrekord Iran

**Keywords:** chicken, fatness, hypoxia, oxidative stress, phytogenic, pomegranate peel

## Abstract

The aim of this study was to evaluate antioxidant, antihyperlipidemic and hypotensive properties of pomegranate peel (PP) on antioxidant status, fat deposition, lipid peroxidation and pulmonary hypertensive response in broiler chickens. A total of 375 one‐day‐old male broilers (Cobb 500) were randomly assigned to five treatments included dietary PP levels of 0, 2.5, 5.0, 7.5 and 10 g/kg. Supplementation of PP at 7.5 and 10 g/kg resulted in significant upregulation of hepatic catalase (*p *< 0.004) and superoxide dismutase1 (SOD1; *p *< 0.05), which reflected in decreased concentration of circulatory malondialdehyde (MDA). Dietary inclusion of PP at 7.5 and 1.0 g/kg significantly decreased serum concentrations of triglycerides (*p *< 0.004) and cholesterol (*p *< 0.006) with concomitant decrease in abdominal fat deposition (*p *< 0.05). The antihyperlipidemic effect of PP was mediated through down‐regulation of peroxisome proliferator activated receptor alpha (PPARα). Hypotensive effect of PP was also observed at 7.5 and 10 g/kg as reduced heart weight and the right‐to‐total ventricular weight ratio (RV/TV) and decreased mortality from pulmonary hypertension. The hypotensive property of PP was associated with increased concentration of serum nitric oxide. In conclusion, this study revealed antioxidative, antihyperlipidemic and hypotensive effects of PP at 7.5 and 10 g/kg in broiler chickens exposed to hypobaric hypoxia. Health‐beneficial effects of PP suggest this product as a promising multi‐functional phytogenic feed additive for broiler chickens.

## Abstract 2

In this study, evaluate antioxidant, antihyperlipidemic, and hypotensive properties of pomegranate peel (PP) on antioxidant status, fat deposition, lipid peroxidation, and pulmonary hypertensive response in broiler chickens. Supplementation of PP at 7.5 and 10 g/kg resulted in significant up‐regulation of hepatic catalase, superoxide dismutase1 and peroxisome proliferator activated receptor alpha (PPARα), which reflected in decreased concentration of circulatory malondialdehyde (MDA).

## 1 INTRODUCTION

Modern broiler chickens have been extensively selected for rapid growth rate and bigger mass of breast meat. This has resulted in higher metabolic rate, which favours oxidants (i.e. increased production of the reactive oxygen species (ROS) over the antioxidant capacity of birds (Frisard & Ravussin, 2006; Khajali & Wideman, 2016). Commercial production of broilers exacerbates oxidative stress by virtue of exposing birds to long photoperiods, high level of dust, ammonia, etc. A great deal of information indicates that oxidative stress severely impacts poultry performance and health (Rafiei & Khajali, 2021; Surai et al., 2019).

Broiler chickens accumulate a high degree of polyunsaturated fatty acids (PUFA) in meat lipids and the abdominal cavity (Cui et al., 2012). This is a potential factor that exacerbates oxidative stress because double bonds are the target of ROS (Ahmadipour, Sharifi, et al., 2018; Ahmadipour, Hassanpour, et al., 2018). Oxidative stress has been known to be the principal trigger to the development of pulmonary hypertension syndrome (PHS; also known as ascites) in broiler chickens (Arab et al., 2006; Cawthon et al., 1999; Khajali & Wideman, 2016). High altitude limits oxygen availability and exacerbates oxidative stress by intensifying ROS production and results in faster developing PHS (Balog, 2003). Adverse effect of oxidative stress can be attenuated by inclusion of adequate supply of antioxidants in broiler nutrition. Antioxidant system controls ROS production and maintain the redox (antioxidant/pro‐oxidant) balance.

Public choice toward the use of natural products has led to the use of natural feed additives such as natural antioxidants in poultry nutrition. Plant secondary metabolites exert a wide range of health benefits including antioxidant properties. Polyphenols are the biggest phytochemicals with strong antioxidant property (Gessner et al., 2017). Polyphenols also possess several beneficial properties such as antihyperlipidemeic, bactericidal and hypotensive properties (Neyrinck et al., 2013).

Pomegranate peel (PP), obtained from *Punicagr anatum*, often lefts over from production of the juice. It possesses potential health benefits due to a rich content of antioxidant polyphenols. Pomegranate peel contains the highest contents of polyphenols and flavonoids and the greatest antioxidant capacity compared to the seed and juice (Derakhshan et al., 2018). Sharifian et al. (2019) reported that dietary supplementation with PP extract at 250, 450 and 650 mg/kg linearly reduced the MDA concentration in breast muscle of broiler chickens under heat stress. A review of literature further corroborated that dietary inclusion of PP extract up to 300 mg/kg significantly increased total phenolic contents and antioxidant activity in the breast meat of broiler chickens (Rafiei & Khajali, 2021). An extract of PP has sometimes been added to fruit juices to increase shelf‐life of juices (Salgado et al., 2012). Furthermore, PP has been used to improve the shelf life of chicken meat due to antioxidant and antimicrobial effects (Kanatt et al., 2010). In the present study, we used PP as a phytogenic feed additive to investigate its effects on productivity, antioxidant status, hepatic lipogenesis and occurrence of PHS in broiler chickens reared at high land (2100 m).

## 2 MATERIALS AND METHODS

### 2.1 Birds and experimental

The study was carried out in an area with an altitude of 2100 m above sea level. A total of 375 male day‐old broiler chicks (Cobb, 500) were selected from a population of 375 to have as narrow weight range as possible and they were randomly divided to 25 groups (floor pens with 2.8 m × 2.8 m) to have 15 birds per pen. Five pens were randomly assigned to each of five treatments. Birds raised for 40 days in a standard condition outlined by the manufacturer (Cobb 500 guideline).

### 2.2 Treatments

A basal diet was prepared according to the Cobb 500 recommendations for broiler chickens and regarded as the control. The basal diet was then supplemented with 2.5, 5.0, 7.5 and 10 g/kg PP to make treatment groups. In the present study, pomegranate was collected from Ardal district, located in CHB province of Iran. The peels were manually removed, air‐dried and finely ground for use in the experimental diets. Chemical composition of PP was assayed and presented in Table 1. Experimental diets were formulated to have similar metabolisable energy and protein contents (Table 2) and offered in mash form.

**Table 1:** Chemical composition and bioactive compounds of pomegranate peel extract

Compound	Content (mg/g)
Dry matter	976
Crude protein	83
Crude fibre	213
Ether extract	29
Ash	51
Total phenolic compounds	331
Proanthocyanin	33.6
Punicalagin	26.3
Ellagic acid	1.12
Oleuropein	2.18
Gallic acid	3.38
Caffeic acid	0.93
Catechin	0.63

**Table 2:** Composition of the basal diet fed to broilers chickens from 1 to 40 days of age

Item (g/kg unless noted)	Starter (1–10 days)	Grower (11–20 days)	Finisher (21–40 days)
Corn	563.5	610.0	626.0
Soybean meal (44% CP)	347.0	303.0	278.0
Soy oil	35.0	33.5	45.0
Dicalcium phosphate	17.0	17.0	16.0
Oyster shell	13.0	12.0	11.0
Salt	4.0	4.0	4.0
DL‐methionine	3.5	3.5	3.5
L‐lysine	2.0	2.0	1.5
Mineral supplement^1^	2.5	2.5	2.5
Vitamin supplement^2^	2.5	2.5	2.5
Wheat bran^3^	10	10	10
pomegranate peel	–	–	–
AME (kcal/kg)	3000	3085	3170
Crude protein	210.0	190.0	180.0
Met+Cys	9.0	8.9	8.2
Lys	13.3	11.9	10.5
Thr	10.0	8.8	8.2
Arg	13.8	12.5	11.3
(Na^+^+K^+^) – Cl^–^ (mEq/kg)	200	200	200

^1^ Provided the following per kg of diet: Mn (from MnSO_4_‐H_2_O), 40 mg; Zn (from ZnO), 40 mg; Fe (from FeSO_4_‐7H_2_O), 20 mg; Cu (from CuSO_4_‐5H_2_O), 4 mg; I (from Ca(IO_3_)_2_‐H_2_O), 0.64 mg; Se (from sodium selenite), 0.08 mg.

^2^ Provided the following per kg of diet: vitamin A (trans retinyl acetate), 3600 IU; vitamin D3 (cholecalciferol), 800 IU; vitamin E (dl‐α‐tocopheryl acetate), 7.2 mg; vitamin K3, 1.6 mg; thiamine, 0.72 mg; riboflavin, 3.3 mg; niacin, 0.4 mg; pyridoxin, 1.2 mg; cobalamine, 0.6 mg; folicacid, 0.5 mg; choline chloride, 200 mg.

### 2.3 Chemical composition

Chemical composition of PP for dry matter, crude protein, crude fibre and crude fat (EE) was determined respectively by 934.01, 992.93, 962.09 and 920.39 methods of Association of Official Analytical Chemists (AOAC, 2010). Total phenolic compounds were extracted by a Suprex MPS/225 system (Pittsburg, USA) and determined using Folin‐Ciocalteau reagent according to Wolfe et al. (2003). In brief, a volume of 0.5 mL of deionised water and 0.125 mL of PP extract were added to a test tube. Folin‐Ciocalteau reagent (0.125 mL) was added to the tube and allowed to stand for 6 minutes. In the next step, 1.25 mL of 7 % sodium carbonate was added to the test tube and the volume brought up to 3 mL by adding deionised water. The absorbance was measured at 760 nm after 90 minutes using a spectrophotometer (Corning 480, Corning, New York, NY, USA). The measurement was compared to a standard curve of prepared gallic acid solutions and expressed as milligrams of gallic acid equivalents per g PP. Ethanolic extract of PP samples was subjected to GC‐MS analysis according to Adams (1995). Chemical reagents used in all assays were obtained from Sigma–Aldrich Co. (Sigma–Aldrich Co., St. Louis, MO, USA).

### 2.4 Measurements

Body weight gain and feed intake were recorded in 1–20, 21–40 and 1–40 days of age periods. Feed conversion ratio (FCR) was also calculated for the same periods. At 40 days, 10 birds were selected from each group for blood collection and processing. The selected birds had body weights within ∼ 5% of the average pen body weight. Blood samples (∼ 3 mL) were collected from brachial vein and centrifuged at 2500 × *g* for 10 minutes to separate sera. Serum samples were tested for total cholesterol (TC), triglycerides (TG) and malondialdehyde (MDA). TC and TG were measured using commercial laboratory kits following the manufacturer's manuals (Pars Azmoon, Tehran, Iran). MDA, as biomarker of lipid peroxidation, was assayed according to Nair and Turner (1984). Nitric oxide (NO) was determined by the method described by Behrooj et al. (2012). Birds were then euthanised by CO_2_ to determine carcass processing including weights of live body, carcass, heart and abdominal fat. The hearts were dissected to calculate the right ventricular‐to‐total ventricular weight ratio (RV:TV). Whenever RV:TV exceeded 0.25, it was considered as ascites (Izadinia et al., 2010; Khajali & Khajali, 2014).

### 2.5 Quantitative real‐time PCR analysis

The liver of 10 birds per treatment was harvested, homogenised and treated by a digestion buffer (RNX‐Plus reagent; Sinaclon Bioscience, Karaj, Iran). An aliquot of 200 μL chloroform was added to the digested samples and centrifuged at 16,000 × *g* for 15 minutes (under cool temperature, 4°C). The supernatant was taken to precipitate RNA using the equal volume of isopropanol. The samples were then rinsed with 75% ethanol, centrifuged and the resulting pellet dissolved in appropriate amount of diethyl dicarbonate (DEPC)‐treated water. The extracted RNA was treated with DNAase kit (Sinaclon Bioscience) to remove residual DNA. The quantity of RNA was checked by O.D. (A260/280) using a biophotometer (Eppendorf, USA). RNA with the absorbance ratio between 1.8 and 2.2 was used for the synthesis of cDNA.

Total RNA was converted to cDNA by means of the PrimeScript™ RT Reagent Kit (Takara Bio Inc., Japan). The levels of superoxide dismutase 1 (SOD1), catalase, peroxisome proliferator‐activated receptor alpha (PPARα) and tyrosine 3‐monooxygenase/tryptophan 5‐monooxygenase activation protein zeta (YWHAZ) transcripts were determined by real‐time RT‐PCR using SYBR® Premix Ex Taq™ II kit (TliRnase H Plus; Takara Bio Inc.). YWHAZ was used as an endogenous reference gene to normalise the input load of cDNA across samples (Hassanpour et al., 2018). Table 3 represents the specific primers of PCR, designed with the online software of Primer‐Blast (www.ncbi.nlm.nih. gov/tools/primer‐blast/index.cgi?LINK_LOC = BlastHome). The amplifications were run in a real‐time PCR cycler (Rotor Gene Q 6000; Qiagen, USA) in three replicates per each liver sample. Data were analysed using LinRegPCR software version 2012.0 (Amsterdam, the Netherlands) to provide the threshold cycle number and reaction efficiency (Ruijter et al. 2009). Relative transcript levels (gene / YWHAZ) were calculated using the efficiency‐adjusted Paffl methodology (Dorak, 2006; Hassanpour et al., 2018).

**Table 3:** Details of the primers used for quantitative real time PCR analysis for chickens

Target	Primers	PCR product (bp)	Accession no.
YWHAZ	F:5′‐AGGAGCCGAGCTGTCCAATG‐3′ R:5′‐CTCCAAGATGACCTACGGGCTC‐3′	84	NM_001031343.1
SOD1	5′‐CACTGCATCATTGGCCGTACCA‐3′ 5′‐GCTTGCACACGGAAGAGCAAGT‐3′	224	NM_205064.1
CAT	F:5′‐TGGCGGTAGGAGTCTGGTCT‐3′ R:5′‐GTCCCGTCCGTCAGCCATTT‐3′	112	NM_001031215.1
PPAR*α*	F:5‐GCTGTGGAGATCGTCCTGGT‐3 R:5‐GTGACAAGTTGCCGGAGGTC‐3	163	NM_001001464.1

YWHAZ = tyrosine 3‐monooxygenase/tryptophan 5‐monooxygenase activation protein zeta; SOD1 = superoxide dismutase 1; CAT = catalase; PPAR*α* = peroxisome proliferator activated receptor alpha; bp = base pair.

### 2.6 Statistical analysis

The ANOVA procedure of SAS (2007) software was adopted to analyse the data in a completely randomised design. Treatment means were separated by the Duncan's multiple range test. Linear, quadratic and cubic effects of dietary PP inclusion levels were studied using orthogonal polynomial contrasts to compare the control with PP groups.

## 3 RESULTS

Table 4 depicts the effect of dietary inclusion of PP on growth performance of broiler chickens. No significant effect was observed among dietary treatments in terms of body weight gain. However, feed intake was linearly reduced by PP inclusion and there was a significant difference between the control and PP included at 7.5 and 10 g/kg (*p* < 0.05). Feed conversion ratio throughout the trial (1–40 days) was improved at 7.5 g/kg PP.

**Table 4:** Effects of different levels of pomegranate peel (PP) on body weight gain (BWG), feed intake (FI), and feed conversion ratio (FCR) in broiler chickens during 1–40 days of age

	Dietary levels of pomegranate peel (g/kg)						*p* Value			
Item	Control (0)	2.5	5	7.5	10	SEM	Control vs. PP^1^	Linear	Quadratic	Cubic
FI (g/b)	3421^a^	3389^a^, ^b^	3305^b^	3268^c^	3236^c^	33.6	0.003	0.0002	0.722	0.599
BWG (g/b)	1807	1842	1817	1842	1768	22.7	0.169	0.293	0.063	0.608
FCR	1.89^a^	1.84^a^, ^b^	1.82^a^, ^b^	1.77^b^	1.83^a^, ^b^	0.024	0.062	0.032	0.056	0.425

^a^ Means in the same raw with different letter superscripts are significantly different.

^b^ Means in the same raw with different letter superscripts are significantly different.

^c^ Means in the same raw with different letter superscripts are significantly different.

^1^ Orthogonal contrast.

Table 5 indicates serum variables of broilers received different levels of PP in the feed. Regression analysis indicated a significant linear response for serum variables when PP included in broiler diets. Broilers received PP at 7.5 and 10 g/kg had significantly (*p* < 0.05) lower concentrations of MDA, TC and TG than the control. However, inclusion of PP at 7.5 g/kg significantly increased circulatory level of NO compared to the control. They also deposited less fat in the abdomen compared to their counterparts in the control group. Orthogonal contrast between the control vs. PP groups was significant for serum variables.

**Table 5:** Effect of different levels of pomegranate peel (PP) on serum variables and abdominal fat deposition in broiler chickens measured at 40 days of age

	Dietary levels of pomegranate peel (g/kg)						*p* Value			
metabolites	Control (0)	2.5	5	7.5	10	SEM	Control vs. PP^1^	Linear	Quadratic	Cubic
Malonedialdehyde (μmol/L)	3.68^a^	3.3^a^, ^b^	2.9^a^, ^b^	2.7^b^	2.5^b^	0.3	0.069	0.004	0.576	0.868
Nitric oxide (μmol/L)	11.9^b^	13.2^a^, ^b^	14.3^a^, ^b^	16^a^	16.5^a^	1.05	0.021	0.0009	0.791	0.788
Triglycerides (mg/dL)	179.7^a^	162.6^a^, ^b^	155.2^b^	141^b^	143.5^b^	7.3	0.004	0.0006	0.051	0.562
Cholesterol (mg/dL)	114.6^a^	111.3^a^	107.3^a^, ^b^	97.2^c^	99.1^b^, ^c^	3.00	0.006	0.0001	0.689	0.047
Abdominal fat (%)	1.85^a^	1.83^a^	1.73^a^, ^b^	1.48^b^	1.51^b^	0.108	0.050	0.002	0.679	0.259

^a^ Means in the same raw with different letter superscripts are significantly different.

^b^ Means in the same raw with different letter superscripts are significantly different.

^c^ Means in the same raw with different letter superscripts are significantly different.

^1^ Orthogonal contrast.

[Correction added on 23 June 2021, after first online publication: The dietary levels of pomegranate peel (g/kg) were corrected to “2.5, 5, and 7.5”, to be consistent with values in the other tables.]

The effects of PP supplementation on carcass characteristics and ascites mortality of broiler chickens are shown in Table 6. Dietary supplementation with PP at 7.5 g/kg reduced heart weight, and RV/TV when compared to the control. Cumulative ascites mortality was significantly (*p *= 0.001) reduced when dietary inclusion of PP exceeded 5 g/kg. Orthogonal contrasts of RV:TV between the control vs. PP groups was significant (*p*  = 0.001; df = 1). It is evident that dietary PP inclusion significantly prevents right ventricular hypertrophy (as reflected in lower RV/TV values) and PHS mortality in a linear manner.

**Table 6:** Effect of different levels of pomegranate peel (PP) on carcass characteristics and ascites mortality of broiler chickens raised up to 40 days of age

	Dietary levels of pomegranate peel (g/kg)						*p* Value			
Item	Control (0)	2.5	5	7.5	10	SEM	Control vs. PP^1^	Linear^2^	Quadratic	Cubic
Carcass (% LBW)	71.1	71.7	72.1	72.3	72.1	0.51	0.512	0.116	0.389	0.911
Heart (% LBW)	0.611^a^	0.585^a^, ^b^	0.564^a^, ^b^	0.538^b^	0.548^b^	0.016	0.044	0.003	0.324	0.587
RV (% LBW)	0.120^a^	0.110^a^, ^b^	0.108^a^, ^b^	0.102^a^, ^b^	0.095^b^	0.007	0.035	0.005	0.560	0.137
TV (% LBW)	0.394	0.399	0.410	0.417	0.382	0.016	0.399	0.746	0.164	0.184
RV:TV	0.307^a^	0.298^a^	0.257^a^, ^b^	0.243^b^	0.238^b^	0.017	0.015	0.0009	0.588	0.477
Cumulative ascites mortality (%)	14.6^a^	12.0^a^, ^b^	8.0^b^, ^c^	6.6^c^	6.6^c^	1.4	0.001	0.0001	0.144	0.561

^a^ Means in the same raw with different letter superscripts are significantly different.

^b^ Means in the same raw with different letter superscripts are significantly different.

^c^ Means in the same raw with different letter superscripts are significantly different.

RV:TV, right ventricle to total ventricle weight ratio; LBW, live body weight.

^1^ Orthogonal contrast.

The expression of PPARα, SOD1 and catalase genes in the liver of broilers was affected by dietary PP (Table 7). PPARα, catalase and SOD1 genes were linearly overexpressed in broilers fed PP at 7.5 g/kg relative to the control.

**Table 7:** Effect of different levels of pomegranate peel (PP) supplements on hepatic gene expression of broiler chickens

	Dietary levels of pomegranate peel (g/kg)						*p* Value			
Gene	Control (0)	2.5	5	7.5	10	SEM	Control vs. PP^1^	Linear	Quadratic	Cubic
PPAR*α*	0.0072^b^	0.0100^b^	0.0260^a^, ^b^	0.0680^a^	0.0784^a^	0.015	0.023	0.001	0.560	0.449
CAT	0.0006^b^	0.0007^b^	0.0073^b^	0.1351^a^	0.1286^a^	0.04	0.029	0.004	0.481	0.278
SOD1	0.0065^b^	0.0133^b^	0.160^a^, ^b^	0.290^a^	0.303^a^	0.088	0.052	0.003	0.988	0.367

^a^ Means in the same raw with different letter superscripts are significantly different.

^b^ Means in the same raw with different letter superscripts are significantly different.

^c^ Means in the same raw with different letter superscripts are significantly different.

PPAR*α*, peroxisome proliferator activated receptor alpha; CAT, catalase; SOD1, superoxide dismutase 1.

^1^ Orthogonal contrast.

## 4 DISCUSSION

Reduced feed intake of birds when PP included at 7.5 and 10 g/kg could be expected, and it is associated with high crude fibre content of PP. Improved FCR in birds fed with 7.5 g/kg PP could be explained by reduced feed intake and compromised weight gain. This finding is in line with those of Sarica and Urkmez (2016) who reported improved FCR when broiler diets supplemented with ethanolic extract of PP.

Dietary supplementation with PP lowered circulatory levels of TC and TG. This finding indicated antihyperlipidemic properties of PP. It has been reported that feeding PP extract to broiler chickens significantly reduced serum LDL cholesterol and TG levels though significantly increased serum HDL cholesterol levels compared to the control (Sarıca & Urkmez, 2016). Reduced accumulation of fat in abdominal cavity in birds fed PP further confirms this observation. The lipid‐lowering effect of PP may be regulated through upregulation of PPAR‐α. PPAR‐ α is a transcription factor and it is a major regulator of lipid metabolism in the liver (Ahmadipour, Sharifi, et al., 2018; Fruchart, 2009). PPAR‐ α activation has shown to decrease triglycerides (TG) by increasing fatty acid β‐oxidation and hepatic lipoprotein lipase expression (Fruchart, 2009). Aviram and Fuhrman (2001) also reported that pomegranate extract attenuated low‐density lipoprotein oxidation. In birds, the liver is the main site of lipogenesis (Steven, 1996). Esmaillzadeh et al. (2006) reported that pomegranate extract decreased total cholesterol and LDL‐cholesterol by modulating the activity of HMG‐CoA reductase. In agreement with our results, Xu et al. (2009) demonstrated over‐expression of PPAR*α* by feeding pomegranate extract to rats.

Birds received PP at 7.5 and 10 g/kg had significantly lower heart weight and RV:TV. The RV:TV ratio is an index of right ventricular hypertrophy and reflects pulmonary hypertension (Ahmadipour, Hassanpour, et al., 2018). These results clearly indicate hypotensive effect of PP in broiler chickens, which could be associated with bioactive components of PP. Punicalagin, a bioactive component of PP, has shown to modulate vascular damage resulting from oxidative stress in hypoxic pulmonary hypertension (Shao et al., 2016). Furthermore, Pomegranate has been reported to induce endothelial nitric oxide synthase gene regulation in human coronary endothelial cells (Stowe, 2011). In the present study, serum NO concentration was significantly increased when PP included at 7.5 and 10 g/kg. This observation suggests the hypotensive effect of PP mediated through changes in circulatory level of NO. Khajali and Wideman (2010) have reviewed the role of NO in prevention of pulmonary hypertension in broiler chickens. Consequently, mortality from pulmonary hypertension has been significantly decreased in the corresponding treatment groups compared to the control (6.6 vs. 14.6%).

Inclusion of PP at 7.5 and 10 g/kg was associated with reduced level of MDA. MDA is an index of lipid peroxidation and oxidative damage caused by ROS. ROS are highly influential factor in the pathogenesis of pulmonary hypertension (Zuo et al., 2014). Although ROS formation naturally occurs through various metabolic processes in cells, excessive amount of ROS is detrimental to the cell and subsequently the entire organism. This study revealed that PP is a strong free radical scavenger because of its antioxidant constituents such as polyphenols and anthocyanins. It has been shown that total phenolics content of PP extract was nearly 10‐fold as high as that of pomegranate pulp extract (Li et al., 2006). This finding clearly indicates that PP contains more antioxidants than does flesh tissue of pomegranate. Antioxidant properties of PP to prevent lipid peroxidation (as reflected in reduced MDA level) may be associated with over‐expression of antioxidant genes including Catalase and SOD1. PP at 7.5 g/kg significantly resulted in upregulation of catalase and SOD1. In line with this finding, Hasan et al. (2020) reported that Supplementing pomegranate extract to rabbit diet at 100, 150 and 200 mg/kg significantly increased the activity of SOD and GSH‐Px compared to the control group.

## 5 CONCLUSION

Supplementation of PP at 7.5 g/kg could result in reduced hepatic lipogenesis through upregulation of PPRAα. Dietary inclusion of PP at 7.5 g/kg prevents lipid peroxidation and pulmonary hypertension in broiler diets. PP seems to be a promising natural antioxidant to be included in broiler diets.

## AVAILABILITY OF DATA AND MATERIALS

Date are ready upon request.

## AUTHOR CONTRIBUTIONS

Conceptualisation: BA. Data curation: SP and SA. Formal analysis: HH, FK. Investigation: FK, BA. Methodology, BA, FK. Project Administration: SA. Writing manuscript: FK, BA.

All authors contributed to the revision of the manuscript and approve of the final version of the article.

## CONFLICTS OF INTEREST

The authors declare that there are no conflicts of interest.

## ETHICS STATEMENT

The Ethical Committee of Shahrekord University Research Council approved all procedures used in the study in accordance with the standard of 1964 Declaration of Helsinki.

### PEER REVIEW

The peer review history for this article is available at https://publons.com/publon/10.1002/vms3.556.
